# Identification of Genes Required for Alternative Oxidase Production in the *Neurospora crassa* Gene Knockout Library

**DOI:** 10.1534/g3.112.004218

**Published:** 2012-11-01

**Authors:** Frank E. Nargang, Kelly Adames, Cornelia Rüb, Serena Cheung, Nancy Easton, Cheryl E. Nargang, Michael S. Chae

**Affiliations:** Department of Biological Sciences, University of Alberta, Edmonton, Alberta, Canada, T6G 2E9

**Keywords:** alternative oxidase, knockout library, *Neurospora crassa*, mitochondria

## Abstract

The alternative oxidase (AOX) of *Neurospora crassa* transfers electrons from ubiquinol to oxygen. The enzyme is not expressed under normal conditions. However, when the function of the standard electron transport chain is compromised, AOX is induced, providing cells with a means to continue respiration and growth. Induction of the enzyme represents a form of retrograde regulation because AOX is encoded by a nuclear gene that responds to signals produced from inefficiently functioning mitochondria. To identify genes required for AOX expression, we have screened the *N. crassa* gene knockout library for strains that are unable to grow in the presence of antimycin A, an inhibitor of complex III of the standard electron transport chain. From the 7800 strains containing knockouts of different genes, we identified 62 strains that have reduced levels of AOX when grown under conditions known to induce the enzyme. Some strains have virtually no AOX, whereas others have only a slight reduction of the protein. A broad range of seemingly unrelated functions are represented in the knockouts. For example, we identified transcription factors, kinases, the mitochondrial import receptor Tom70, three subunits of the COP9 signalosome, a monothiol glutaredoxin, and several hypothetical proteins as being required for wild-type levels of AOX production. Our results suggest that defects in many signaling or metabolic pathways have a negative effect on AOX expression and imply that complex systems control production of the enzyme.

Although the proper formation of mitochondria requires the expression of genes found in both the nuclear and mitochondrial genomes, the vast majority of mitochondrial proteins are encoded in the nucleus. Thus, communication of mitochondrial status to the nucleus must occur to insure that the organelles meet the requirements for growth and development of the organism and to insure that they can properly respond to changing conditions and stress. Communication from the mitochondria to the nucleus that influences the expression of nuclear genes has been termed retrograde regulation (Jazwinski and Kriete 2012; Liu and Butow 2006; Woodson and Chory 2008).

The alternative oxidase (AOX) is a di-iron carboxylate protein that transfers electrons from ubiquinol to molecular oxygen and exists in the mitochondrial inner membrane (Albury *et al.* 2009; Andersson and Nordlund 1999; Berthold *et al.* 2000; Berthold and Stenmark 2003). It is found in a variety of organisms, including bacteria, protists, fungi, plants, and animals—but not mammals (McDonald 2008; McDonald and Vanlerberghe 2006). Depending on the organism, expression of AOX can be influenced by developmental signals, tissue specificity, and response to stress (Considine *et al.* 2001; Djajanegara *et al.* 2002; Finnegan *et al.* 1997; Karpova *et al.* 2002; Nargang and Kennell 2010; Van Aken *et al.* 2009; Vanlerberghe and McIntosh 1997). In many organisms, AOX occurs at low-to-undetectable levels under normal growth conditions but becomes highly expressed when the standard, cytochrome-mediated, electron transport chain (sETC) is compromised. Thus, because AOX is encoded in the nucleus, it serves as a prime example of a gene that is controlled by retrograde regulation. However, the nature of the retrograde pathway(s) and the factors required to achieve regulation of AOX production are not well understood.

In fungi, a few genes are known that affect AOX production. In *Candida albicans* there is evidence for the involvement of a histidine kinase in AOX regulation (Huh and Kang 2001). Early work with *Neurospora crassa* identified the structural gene for AOX as *aod-1* whereas another gene, *aod-2*, was found to be required for the expression of *aod-1* (Bertrand *et al.* 1983; Edwards *et al.* 1976; Lambowitz *et al.* 1989; Li *et al.* 1996). More recently, we used a reporter system and a traditional genetic screen to identify four additional genes, named *aod-4*, *aod-5*, *aod-6*, and *aod-7*, along with a new allele of *aod-2*, as required for AOX induction (Descheneau *et al.* 2005). Thus, these studies defined a minimum of five genes required for proper AOX production in *N. crassa*. We then demonstrated that *aod-2* and *aod-5* encode transcription factors (Chae *et al.* 2007b) of the Zn(II)_2_Cys_6_ binuclear cluster (zinc cluster) family (MacPherson *et al.* 2006). *In vitro* studies suggested that the proteins form a heterodimer, which binds a specific sequence within the *aod-1* promoter region to activate transcription under the appropriate inducing conditions (Chae *et al.* 2007a,b; Chae and Nargang 2009). Orthologs of AOD2 and AOD5 also are required for AOX production in *Podospora anserina* (Sellem *et al.* 2009) and *Aspergillus nidulans* (Suzuki *et al.* 2012).

Because the previously described mutant screen was not saturated, it seemed likely that additional genes might also be involved with AOX regulation. However, two factors led us to not simply repeat the screen. First, the screen was designed to detect mutations affecting transcriptional regulation of the *aod-1* gene. Additional factors affecting posttranscriptional processes that also may play a role in expression of AOX would not be detected. Second, a gene knockout library for *N. crassa* (Colot *et al.* 2006) has been created since our previous screen. Identification of strains affected in their ability to produce AOX in this library would allow direct identification of the genes without the need for mapping and rescue experiments. Here we describe 62 newly identified genes from the *N. crassa* knockout library that affect the production of AOX to varying extents.

## Materials and Methods

### Strains and growth of *N. crassa*

The *N. crassa* gene knockout library (Colot *et al.* 2006) was obtained from the Fungal Genetics Stock Center (FGSC) in a series of 96-well microtiter plates holding conidia from individual strains in each well (McCluskey *et al.* 2010). At the end point of the study described herein, plates 1 through 108 of the library had been examined. Strain 74*A*-OR23-1A (Nargang lab name NCN251) was used as a control for experiments with the knockout strains.

The *tom20^RIP^* sheltered heterokaryon, the methods used to manipulate the heterokaryon, and the control (strain HIV) for experiments with the heterokaryon have been described in detail previously (Harkness *et al.* 1994a). In summary, the *tom20^RIP^* sheltered heterokaryon contains two nutritionally complementing nuclei. One of the nuclei carries a functional *tom20* gene, a mutation in the *pan-2* gene, and is sensitive to p-fluorophenylalanine (fpa). The other nucleus contains a nonfunctional *tom20* allele that was destroyed by repeat-induced point mutation (RIP) mutagenesis. The latter nucleus also carries a mutant allele of the *mtr* gene that imparts resistance to fpa, as well as a mutation in the *his-3* gene. The level of the Tom20 protein can be greatly reduced in the heterokaryon by growth in the presence of fpa plus histidine.

*N. crassa* cells were grown on solid or in liquid medium according to previously described methods (Davis and De Serres 1970) but using the modified Vogel’s salts developed by Metzenberg (Metzenberg 2004). Medium containing sorbose was used when colonies were desired whereas sucrose containing medium was used when filamentous growth was desired. When needed, inhibitors were added to media at the following final concentrations: antimycin A, 0.5 µg/mL (0.9 µM); chloramphenicol (Cm), 2 mg/mL (6 mM); fpa, 400 µM as described previously (Descheneau *et al.* 2005; Nargang *et al.* 1995). At the aforementioned concentration, antimycin A completely inhibits the sETC.

For screening the *N. crassa* knockout library, a 48-pin inoculation stamp was used that fit precisely into one half of the wells of the 96-well microtiter plates containing the library. Conidia from the library were stamped to Petri plates containing Vogel’s sorbose medium plus antimycin A and to Petri plates without the inhibitor as a control for growth under normal conditions. Petri plates containing antimycin A were examined 5−7 d after inoculation, and those without antimycin were examined 2−3 d after inoculation.

Growth rates in the presence or absence of antimycin A were measured in two ways. In some cases, filamentous growth was monitored by measuring the radius of growth from conidia inoculated in the center of a Petri dish containing Vogel’s sucrose medium at selected time points. In other cases the amount of growth of colonies produced on sorbose containing medium after 2 to 5 d of growth was observed.

### Mitochondrial protein import assays

The procedures used to measure the import of mitochondrial precursor proteins have been described previously (Harkness *et al.* 1994b). In summary, radiolabeled precursors, synthesized by *in vitro* translation in the presence of [^35^S]-methionine, were incubated with mitochondria isolated from freshly grown mycelium. After import for specified time periods, proteinase K was added to remove unincorporated precursor proteins. Mitochondria were washed, and mitochondrial proteins were subjected to polyacrylamide gel electrophoresis in the presence of sodium dodecyl sulfate (SDS-PAGE). Proteins were transferred to nitrocellulose and exposed to X-ray film.

### Other techniques

Standard procedures were used for gel electrophoresis of proteins (Lämmli 1970), western blot analysis (Good and Crosby 1989), and isolation of mitochondria (Nargang and Rapaport 2007). Measurement of the effect of inhibitors on oxygen consumption in growing cells using an oxygen electrode was as described (Tanton *et al.* 2003). Potassium cyanide (CN) and salicylhydroxamic acid were used to inhibit the sETC and AOX, respectively. In some figures, irrelevant lanes were removed electronically. Bands on mitochondrial import blots were quantified using Adobe photoshop.

## Results

### The basis of the screen–strains deficient in AOX are unable to grow in antimycin A

**Figure 1:**
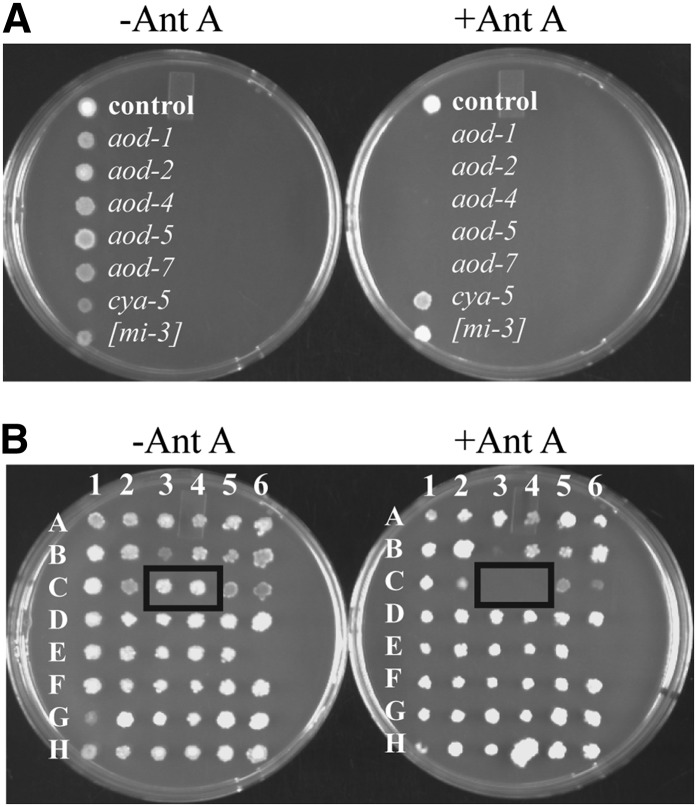
Identification of AOX-deficient strains in the knockout library. (A) Conidial suspensions from strains known to lack AOX (*aod* strains), slow-growing sETC mutants (*cya-5* and [*mi-3*]), and a control wild-type strain (NCN251) were plated on medium with or without antimycin A (+Ant A or –Ant A, respectively). Plates were incubated at 30° for 2 (−Ant A) or 4 (+Ant A) d and then photographed. (B) Plate number 1 of the knockout library contained strains of opposite mating type in which the *aod-5* gene had been knocked out. Conidia from this knockout library plate were stamped onto medium with and without antimycin A. The boxes indicate the position of the *aod-5* strains at positions C3 and C4.

The construction of a library of *N. crassa* strains containing individual gene knockouts (Colot *et al.* 2006) maintained on microtiter plates provided a resource to search for genes that might be involved in the production of AOX. When *N. crassa* is unable to respire through the sETC, it induces AOX, which allows continued growth via this alternative pathway of electron transport. However, if both pathways are blocked, cells are unable to grow. Thus, we reasoned that any of the knockout strains that were unable to grow in the presence of antimycin, which inhibits complex III of the sETC, should also be lacking AOX and the genes affected in such strains would in some way be required for AOX production or function. To test this principle, we set up a microtiter plate containing conidia from several previously described AOX nonproducing strains (Descheneau *et al.* 2005), including a mutant of the *aod-1* gene, which encodes the AOX protein. As controls, we used the parental strain of the knockouts as well as two slow-growing mutants that affected the sETC, *cya-5-34* (Nargang *et al.* 1978), and [*mi-3*] (Mitchell *et al.* 1953). A 48-pin stamp was used to transfer conidia from the microtiter plate to Petri dishes containing growth medium with and without antimycin A. Growth of the control and sETC mutant strains in the presence of antimycin A was obvious whereas AOX mutants failed to grow (Figure 1A). As a further test, we stamped out the section of the knockout library known to contain the two different mating type strains with a knockout of the *aod-5* gene (plate 1, co-ordinates C3 and C4), which we had previously identified as a transcription factor necessary for the expression of AOX (Chae *et al.* 2007b). Of the 47 strains that grew on the control plate without antimycin A, only the two containing the *aod*-5 knockout did not grow in the presence of antimycin A (Figure 1B).

### Examining the knockout library for strains unable to grow on antimycin A

We then proceeded to test the complete library, which consisted of approximately 10,000 strains contained on 108 microtiter plates (96 wells each). Because many of the knocked-out genes are represented in duplicate as both *A* and *a* mating type strains, the actual number of knocked out genes screened was approximately 7800. In the initial screening we identified 218 colonies that grew very slowly, or not at all, on medium containing antimycin A while exhibiting obvious growth on medium lacking the drug. The strains represented by these colonies were then inoculated from the library plates into slants and subsequently retested for growth in Petri dishes containing medium with or without antimycin A. In this round of testing, several strains showed relatively good growth in the presence of the drug. The difference in results compared to the original growth observed in the stamped library might be due to low numbers of viable conidia in some wells of the library plates. Other strains were found to grow extremely slowly even on medium that lacked antimycin A. Strains in either of these two categories were not examined further leaving 126 strains with slow, or no growth, in the presence of antimycin A and reasonably robust growth in the absence of the drug.

To continue our screen, we wished to determine the level of AOX that could be produced in each of the remaining 126 mutants. Because the strains were selected based on inability to grow in the presence of antimycin A, we were unable to use the same drug as an AOX inducer for experiments requiring growing cells. Growth in the presence of Cm at 2 mg/mL results in severe but not total inhibition of synthesis of mitochondrially encoded subunits of the respiratory chain and ATP synthase and results in AOX induction in wild-type cells of *N. crassa* (Li *et al.* 1996; Tanton *et al.* 2003). However, we have previously shown that cells unable to produce AOX grow at rates virtually indistinguishable from the wild type in the presence of Cm (Descheneau *et al.* 2005). We assume that the small amount of mitochondrial protein synthesis that occurs in the presence of Cm is sufficient to support growth of the cells. However, the effects on mitochondria are sufficient to elicit AOX induction.

**Figure 2:**
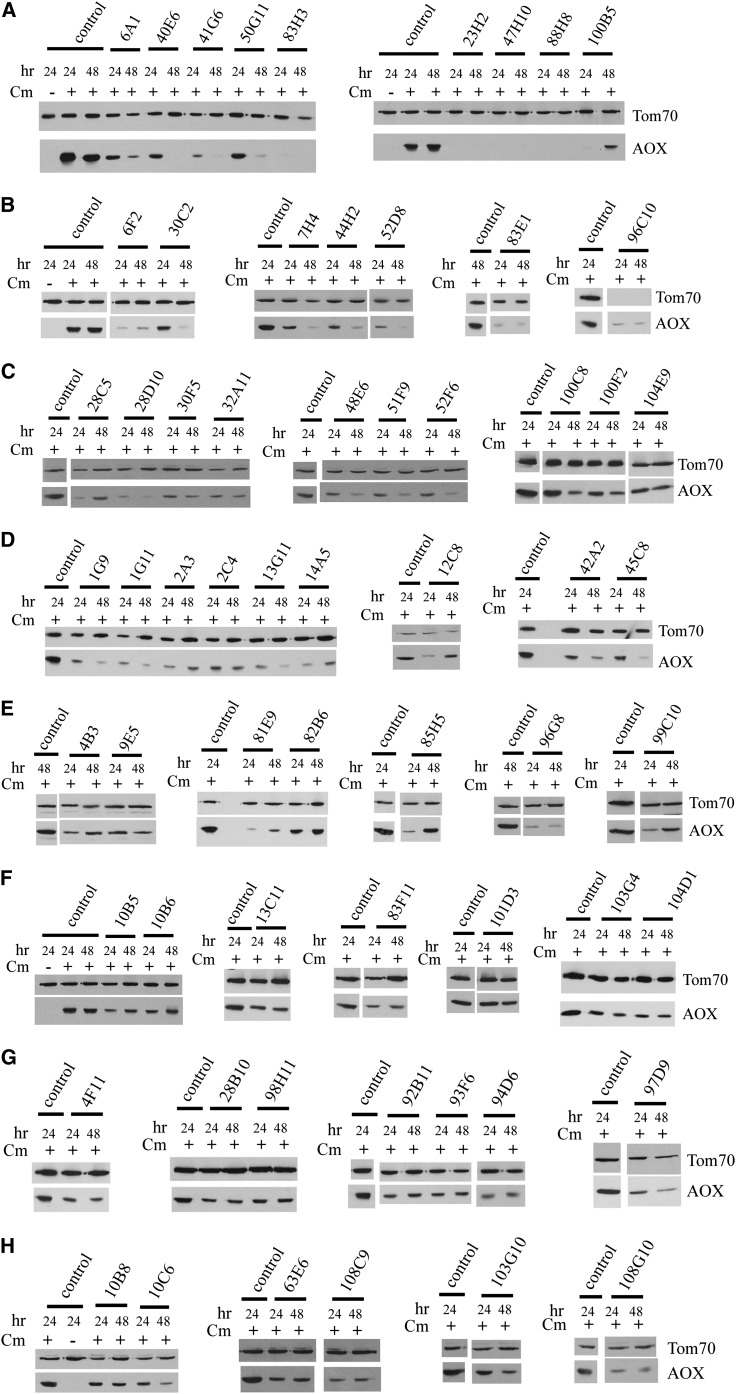
AOX levels in knockout mutants identified in the screen. The indicated strains were grown for 24 and 48 hr in the presence of Cm. Mycelia were harvested, mitochondria were isolated, and mitochondrial proteins were subjected to SDS-PAGE and Western blot analysis using antibody to AOX, and to Tom70 as a loading control. Results are placed in rows A through H to facilitate reference to results for individual strains. The control (NCN251) was grown in similar fashion. A culture of the control without Cm also was used to demonstrate the lack of AOX without induction by Cm. AOX-deficient strains are shown with a control from the same Western blot. In some cases, irrelevant lanes were removed from the blots and are shown as white strips between lanes.

After growth for 24 hr and 48 hr in the presence of Cm, mycelia were harvested and mitochondria were isolated. Western blot analysis of mitochondrial proteins revealed that 62 newly identified strains contained reduced levels of AOX at one or both of the time points (Figure 2). Because the NCU numbers of the predicted proteins at the Neurospora database can change on the basis of more defined annotations, the coordinates of the knockout library 96-well microtiter plates have been used as the primary name for the strains identified. We have used the convention of: number of plate (1−108), followed by the letter of the row (A-H), followed by the number of the column (1−12). Based on a visual assessment of the amount of AOX present on Western blots, we placed each strain into one of three classes (Table 1). Class 1 strains produced virtually no AOX at either 24 hr or 48 hr, or at both time points. An example is strain 23H2 (Figure 2A). Table 1 shows 11 Class 1 strains, but three of these were previously described as AOX-deficient mutants (1C3, *aod-5*; 96H9, *aod-1*; 97B1, *aod-2*). Thus, there are eight new strains that have been identified and assigned to Class 1. The 20 strains in Class 2 exhibited a moderate reduction in the amount of AOX at 24 hr, 48 hr, or both time points. An example is strain 6A1 (Figure 2A). Class 3 contained 34 strains that exhibited some reduction in AOX at one or both of the time points. Strain 4B3 serves as an example (Figure 2E). The NCU number of the predicted proteins and the FGSC strain number of the knockout strain are also shown in Table 1. The BROAD genome sequence website was examined for each gene identified. Known gene functions or characteristic protein domains listed at that website are also included in Table 1. In addition, we have included information regarding the growth rate of the strains in the presence and absence of antimycin A as well as observations on the ability of the strains to form conidiaspores. For unknown reasons, despite the fact that we screened for mutants with poor growth in the presence of antimycin A, a few strains were ultimately found to have good growth in the presence of the drug.

**Table 1:** Summary and characterization of knockout strains identified as AOX deficient

Knockout Library Grid Number	Mutant Class	Panel of Figure 2 Showing Western	NCU Number*^a^*	FGSC Number	Known or Predicted Protein or Gene*^a^* (Domain Identified)	Growth Defect*^b^*	Growth on Antimycin A for48 hr*^c^*	Conidiation Defect*^d^*
1C3*^e^*	1	na	03938.5	11227	*aod-5*		0	
23H2	1	A	08887.5	15957	Hypothetical protein (major facilitator superfamily)		0	
40E6*^f^*	1	A	05600.5	13805	Ubiquinone biosynthesis protein (ABC1 family)		0	
			12096.5		Aspartyl aminopeptidase (glycosyl hydrolase family)			
41G6	1	A	03589.5	13923	Hypothetical protein	Slight	0	Slight
47H10	1	A	00778.5	16938	sed5 vesicle protein (transmembrane adaptor Erv26)	Severe	0	Severe
52D8	1	B	07281.5	14469	Glucose-6-phosphate isomerase (phosphoglucose isomerase)	Severe	0	Slight
83H3	1	A	08365.5	18277	RNA polymerase II mediator complex component Med8	Slight	0	Severe
88H8	1	A	01542.5	19221	Hypothetical protein (HbrB like domain)		0	
96H9*^e^*	1	na	07953.5	18947	*aod-1*			
97B1*^e^*	1	na	03352.5	19465	*aod-2*			
100B5	1	A	08158.5	19644	Dual-specificity phosphatase	Slight	0	Slight
1G9	2	D	00157.5	11281	COP 9 signalosome-1, *csn-1*	Slight	0	Slight
1G11	2	D	00467.5	11283	*csn-5*	Slight	0	Slight
2A3	2	D	08741.5	11299	Striatin pro 11 (WD domain, G-beta repeat)		0.7	Severe
6A1	2	A	06799.5	11001	Fungal specific transcription factor, *vad-5*		0	
6F2	2	B	09739.5	11062	Hypothetical protein, *ada-7* (Zn(2)-Cys(6) binuclear cluster)		0.7	Severe
7H4	2	B	03875.5	11780	Chromatin remodeling complex ATPase chain ISW1 (SLIDE, HAND, helicase conserved domain, SNF2 family, type III restriction enzyme, DEAD/DEAH box helicase, class II histone deactylase complex subunits 2 and 3)		0	Severe
12C8	2	D	01266.5	12022	Phosphoinositide-specific phospholipase C		0.6	
13G11	2	D	04566.5	12420	Protein kinase SNF1		0	Slight
14A5	2	D	03727.5	12091	Hyphal anastomosis-2, *ham-2*		0.4	Severe
28C5	2	C	00007.5	16098	pH response regulator palH, *rim-21*		1.0	Slight
28D10	2	C	00593.5	16116	COP9 signalosome-2, *csn-2*	Slight	0.2	Slight
30C2	2	B	06199.5	13193	RNA binding protein Jsn1 (RNA recognition motif)		1.2	Severe
44H2	2	B	04826.5	16834	Hypothetical protein		0	Severe
45C8	2	D	03035.5	13973	Hypothetical protein		0.3	Slight
50G11	2	A	04607.5	14316	Hypothetical protein		0.3	Slight
81E9	2	E	06084.5	20114	Hypothetical protein (Vps51/Vps67)		1.6	Severe
83E1	2	B	07881.5	18239	Hypothetical protein		2.7	Severe
85H5	2	E	09803.5	18375	Thioredoxin (glutaredoxin homolog)	Slight	0	Slight
96C10	2	B	04245.5	18888	Translocase of outer mitochondrial membrane 70, *tom70*		0	Severe
96G8*^g^*	2	E	06727.5	18934	Spermidine 3, *spe-3* (Spermidine synthase)		2.3	
2C4	3	D	03894.5	11324	Serine/threonine protein kinase ste-20		0.2	
4B3	3	E	04062.5	11473	Peroxisome biogenesis factor 20		1.1	
4F11	3	G	08480.5	11671	Hsf-type DNA-binding domain-containing protein		0	Severe
9E5	3	E	08565.5	11945	Hypothetical protein (NACHT domain)		0.4	
10B5	3	F	09212.5	11545	Serine/threonine protein kinase		1.7	
10B6	3	F	06563.5	11546	Serine/threonine protein phosphatase PP2A catalytic subunit (calcineurin-like phosphoesterase)	Slight	0.5	Severe
10B8	3	H	07489.5	11548	Phosphatase-Z-like-1, *pzl-1* (calcineurin-like phosphoesterase)		0.4	Severe
10C6	3	H	03853.5	11559	Peptidyl-prolyl *cis*-trans isomerase (tetratricopeptide repeat)		2.7	Slight
13C11	3	F	06493.5	12370	Guanine nucleotide-binding protein alpha-1 (ADP ribosylation factor)		3.3	
28B10	3	G	08137.5	16091	Hypothetical protein		0	Slight
30F5	3	C	01471.5	13235	Nuclear protein SNF4 (CBS domain)		0	
32A11	3	C	01955.5	13376	Autophagocytosis protein Aut1		1.9	Slight
42A2	3	D	01276.5	16557	N-acetyltransferase 5	Slight	0.5	Slight
					FR47-like protein			
48E6*^g^*	3	C	04771.5	16944	Fructosyl-amino acid oxidase (FAD dependent oxidoreductase)		0.5	Slight
51F9	3	C	09530.5	14398	Hypothetical protein		1.4	
52F6	3	C	08067.5	14491	Osmosensor protein (SH3 domain)		3.5	
63E6	3	H	03802.5	17474	Trimethyllysine dioxygenase (taurine catabolism dioxygenase)		2.6	Slight
82B6	3	E	07112.5	20169	Hypothetical protein (pyridine nucleotide-disulphide oxidoreductase)		1.5	
83F11	3	F	01511.5	18261	Urease accessory protein ureG	Severe	0.5	Severe
92B11	3	G	07579.5	19411	Hypothetical protein (SCA7)		0.2	Severe
93F6	3	G	03708.5	18633	Hypothetical protein (putative methyl transferase)		1.8	
94D6*^g^*	3	G	00712.5	18704	3-hydroxy-3-methylglutaryl-coenzyme A reductase (sterol sensing domain, patched family)		2.6	
97D9	3	G	00360.5	19497	NAD-dependent epimerase/dehydratase		0	Slight
98H11	3	G	10058.5	18976	Phosphoglucomutase 2		1.1	
99C10	3	E	09686.5	20379	Clock-controlled gene-8, *ccg-8* (transcription factor opl1)		0	
100C8	3	C	09208.5	19659	Transcription factor SPT8 (WD domain, G-beta repeat)		0	
100F2	3	C	09527.5	20459	Hypothetical protein		2.0	
101D3	3	F	09560.5	21068	Superoxide dismutase		0	
103G4	3	F	09553.5	21156	3-hydroxybutyryl CoA dehydrogenase		2.2	
103G10*^g^*	3	H	08132.5	21162	Alpha-1,3-glucan synthase, Ags2	Slight	0.5	
104D1	3	F	02973.5	21213	Mitochondrial carrier protein		0.1	
104E9	3	C	01553.5	21233	Para-hydroxybenzoate-polyprenyltransferase, Coq2		0	
108C9	3	H	02979.5	21401	AMP deaminase		1.8	Slight
108G10*^g^*	3	H	08279.5	21450	Hypothetical protein		1.1	

a Information from *N. crassa* database BROAD Institute (http://www.broadinstitute.org/annotation/genome/neurospora/MultiHome.html).

b Growth rates were measured by inoculating conidiaspores at the center of Petri dishes (standard 100 mm × 15 mm size) containing Vogel’s medium. Plates were incubated at 30°. The radius of mycelial extension from the inoculation point was measured (in cm) and compared with the wild-type control strain that covers the surface of the plate (radius of growth from the inoculation point of 4.2 cm) in 48 hr. Strains with a “slight” growth defect were defined as having a growth radius of 1.5 to 4.1 cm in 48 hr. A “severe” growth defect was defined as having a growth radius of less than 1.5 cm in 48 hr.

c Same as *b*, but the medium contained Antimycin A. Measurements of growth (in cm) were taken after 48 hr. The control strain grew 1.7 cm in 48 hr on average under these conditions.

d Formation of asexual conidiaspores was considered slightly defective if conidiation on slants containing 3 mL of Vogel’s medium in a 1 cm × 10-cm test tube was similar to the control but took longer than the control strain. The defect was considered severe if very few conidia formed, even after extended times.

e The *aod-1* (Li *et al.* 1996), *aod-2*, and *aod-5* (Descheneau *et al.* 2005) genes were all identified during the screen and have been described in detail previously.

f Original predicted open reading frame was knocked out but is now known to be divided into two predicted genes.

g Strain maintained in the knockout library as a heterokaryon. This implies that the affected gene is essential for viability.

### Nature of genes defined by annotations and bioinformatics

The genes identified in the screen represent a broad range of functions. Among the 62 new genes, the two most highly represented groups were 16 hypothetical proteins (9E5, 23H2, 28B10, 41G6, 44H2, 45C8, 50G11, 51F9, 81E9, 82B6, 83E1, 88H8, 92B11, 93F6, 100F2, 108G10) and six kinases or phosphatases (2C4, 10B5, 10B6, 10B8, 13G11, 100B5). If the previously identified *aod-2* and *aod-5* genes are included, there are also six predicted transcription factors (1C3, 6A1, 6F2, 97B1, 99C10, 100C8). There is no obvious relationship among the predicted functions of the eight new class 1 genes (Table 1) and in most cases it is not obvious how many of the predicted functions might compromise AOX production. One class 1 gene that might be imagined as influencing AOX expression is the dual specificity phosphatase deleted in 100B5, because kinases and phosphatases are well known as important regulatory enzymes. A complication with respect to class 1 strain 40E6 is that the region replaced in this knockout strain actually covers two genes. One of these (NCU05600.5) is listed as a ubiquinone biosynthesis protein with an ABC1 family domain, whereas the other (NCU12096.5) is an aspartyl aminopeptidase. We suspect that the absence of the gene encoding the putative ubiquinone synthesis protein would be the more likely of the two genes to affect AOX production because the reduced form of ubiquinone is the substrate for AOX. NCU05600.5 has recently been shown to contain an atypical serine-threonine kinase domain and the gene has been named *stk-33* (Park *et al.* 2011). The role of the protein in ubiquinone synthesis is not entirely clear, and its closest *S. cerevisiae* homolog (YPL109c) has not yet been assigned a function. However, the proteins from both species are related to the *S. cerevisiae* YGL119W protein, also known as Coq8 (Do *et al.* 2001). The kinase domain of Coq8 is thought to regulate the activity of Coq3, a ubiquinone biosynthetic enzyme (Tauche *et al.* 2008). However, a coq8 deletion strain has also been shown to have a complex phenotype that includes underproduction of glutathione and sensitivity to oxidative stress (Gazdag *et al.* 2011). Members of the same family of proteins have also been linked to oxidative stress in *Arabidopsis thaliana* (Jasinski *et al.* 2008). It should be noted that a homolog of another ubiquinone synthesis protein, Coq2, was identified as a class 3 mutant (104E9).

Among the 20 class 2 knockouts, it is striking that three encode components of the COP9 signalosome (1G9, 1G11, and 28D10), whereas another two encode predicted transcription factors (6A1, 6F2). The latter two genes were previously identified as *vad-5* and *ada-7*, respectively, in a study of Neurospora transcription factor knockouts (Colot *et al.* 2006). The class 3 genes represent a broad range of predicted function. Four class 3 genes are predicted to act as kinases or phosphatases.

It was of interest to determine how many of the genes identified in our screen encoded proteins that are known or predicted to be localized to mitochondria. An exhaustive compilation that lists proteins actually identified in purified *N. crassa* mitochondria by two-dimensional electrophoresis and mass spectrometry, as well as those predicted to be mitochondrial by various in silico analyses has recently been published (Keeping *et al.* 2011). Each of the NCU numbers identified in our study was compared to the compilation. The matches are shown in Table 2, along with the criteria used in assigning the protein as mitochondrial. Thirteen proteins were found to be known or possible mitochondrial proteins (Table 2).

**Table 2:** Strains in which the gene knockout is predicted to encode a mitochondrially localized protein

Strain	Mutant Class	NCU Number	Localization Criteria*^a^*	Predicted Protein*^b^*
40E6	1	05600	A2	Ubiquinone biosynthesis
96H9	1	07953	C1	Alternative oxidase (AOX)
2A3	2	08741	B1	Striatin pro 11
96C10	2	04245	A1	Tom70 protein import receptor
96G8	2	06727	C1	Spermidine 3, spermidine synthase
9E5	3	08565	B1	Hypothetical protein
13C11	3	06493	D2	Guanine nucleotide binding protein alpha-1
28B10	3	08137	C1	Hypothetical [BLAST yeast Mitochondrial ribosomal protein of the small subunit, has similarity to human mitochondrial ribosomal protein MRP-S36]
82B6	3	07112	A1	Hypothetical [HcaD domain conserved many organisms, NAD(FAD) oxidoreductase]
101D3	3	09560	A1	Superoxide dismutase
103G4	3	09553	A1	3-hydroxybutyryl-CoA dehydrogenase
104D1	3	02973	C1	Mitochondrial carrier protein
104E9	3	01553	C1	Para-hydroxybenzoate-polyprenyltransferase Coq2

a As defined previously (Keeping *et al.* 2011). Category A1 proteins were identified in their study from highly purified mitochondria using mass spectrometry. Category A2 proteins were identified by mass spectrometry in other studies or had other evidence of being located in mitochondria. B1 proteins were identified in an earlier study of the mitochondrial proteome (Schmitt *et al.* 2006) but no other evidence for or against their mitochondrial location exists. C1 proteins were predicted or demonstrated to be mitochondrial by conventional biochemistry. The D2 proteins are listed in MitoP2 Neurospora data, but other evidence that they are not mitochondrial also exists. This refers to a mitochondrial proteome database for several organisms, including *Neurospora* (http://www.mitop.de/).

b Prediction from the *N. crassa* database. Descriptions in brackets after the first description are taken from Keeping *et al.* (Keeping *et al.* 2011).

### Time-dependent AOX expression

When wild-type strains are grown for 24 and 48 hr in the presence of Cm, there is typically no difference in the amount of AOX present in their mitochondria (Figure 2, panel A, left and right blots; panel F, left blot). However, several of the strains identified from the KO library showed obvious differences in AOX abundance at the two different times of growth used in the screen. These observations suggest that different pathways of induction might be activated or used after different times of inhibition of the sETC. Although a few class 3 mutants showed this phenotype, it was most obvious in class 1 and 2, where mutants 1G9, 6A1, 7H4, 13G11, 30C2, 40E6, 41G6, 44H2, 50G11, and 52D8 contained more AOX after 24 hr growth in Cm than at 48 hr. In these cases a positive regulatory factor may become depleted or a negative factor may accumulate. Conversely, mutants 12C8, 14A5, 28C5, 81E9, 85H5, 100B5, and 101D5 contained more AOX after 48 hr than 24 hr. In these mutants a positive factor may accumulate or a negative factor might be depleted.

**Figure 3:**
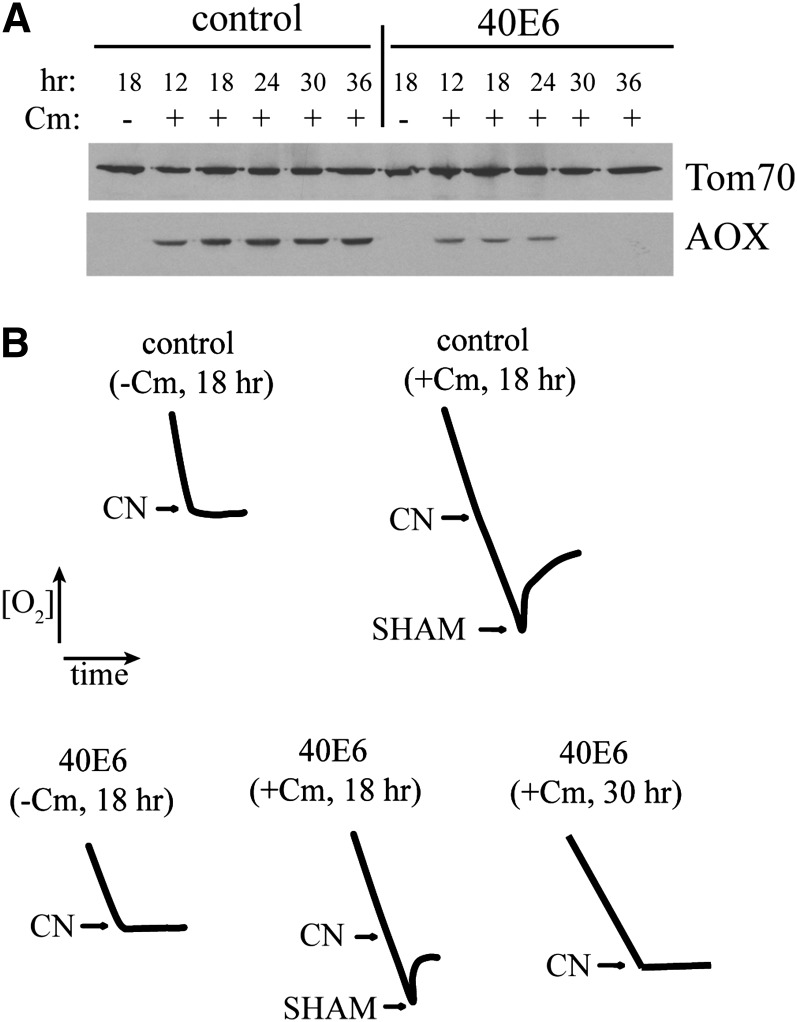
Temporal changes in AOX expression. (A) Mycelium was harvested from cultures of strain 40E6 and the control strain (NCN251) after growth in the presence or absence of Cm for the indicated times. Mitochondria were isolated and subjected to SDS-PAGE and Western blot analysis using the antibodies indicated on the right. (B) Immediately before harvesting the cultures used in (A), 2 mL of the culture was removed and analyzed in a respirometer to determine the effect of inhibitors of the sETC (CN) or the AOX (salicylhydroxamic acid; SHAM) on oxygen consumption. The tracings show the decrease in O_2_ concentration over time. Arrows indicate the point at which the indicated inhibitors were added.

We chose one such class 1 strain (40E6) to determine whether the changes in AOX levels were gradual or sudden. As shown in Figure 3A, the level of AOX in the control strain mitochondria during growth in Cm was consistent over the course of the experiment from 12 to 36 hr. However, for 40E6 grown in Cm, the level of the enzyme was constant from 12 to 24 hr, but then it abruptly disappeared. Furthermore, there was direct correspondence between times that AOX could not be detected by western blot analysis with an absence of the CN-insensitive respiration characteristic of AOX (Figure 3B). It should also be noted that even at the earlier time points, AOX levels are somewhat reduced in 40E6.

### Tom70 acts as the major receptor for the AOX precursor protein

Among the genes identified as affecting AOX production, *tom70* was somewhat unexpected (strain 96C10). Tom70 is a protein of the outer mitochondrial membrane TOM (translocase of the outer mitochondrial membrane) holo-complex (Ahting *et al.* 1999; Dekker *et al.* 1998). The TOM complex is responsible for the import of cytosolically synthesized mitochondrial preproteins into the organelle. Tom70, along with another protein of the complex, Tom20, are the major receptors for mitochondrial precursor proteins in the cytosol (Endo and Yamano 2010; Rapaport and Nargang 2004). Tom70 is chiefly involved in the recognition of hydrophobic proteins of the inner membrane that carry internal targeting signals and multiple membrane spanning domains such as AAC, the ATP/ADP carrier. Tom20 is best known for recognizing matrix-destined precursors carrying an N-terminal targeting signal which is removed from precursor proteins once they reach the matrix. However, Tom20 also has a degree of overlapping specificity for precursors recognized by Tom70 (Brix *et al.* 1999; Söllner *et al.* 1990; Steger *et al.* 1990). Therefore, such proteins are still imported in mutants lacking Tom70, though with reduced efficiency (Grad *et al.* 1999; Steger *et al.* 1990). Because *N. crassa* AOX carries a classic cleavable N-terminal targeting signal (Li *et al.* 1996), it was expected to be imported into mitochondria using Tom20 as its major receptor. However, a few other proteins with cleavable targeting signals have been shown to depend on Tom70 for their import (see *Discussion*).

**Figure 4:**
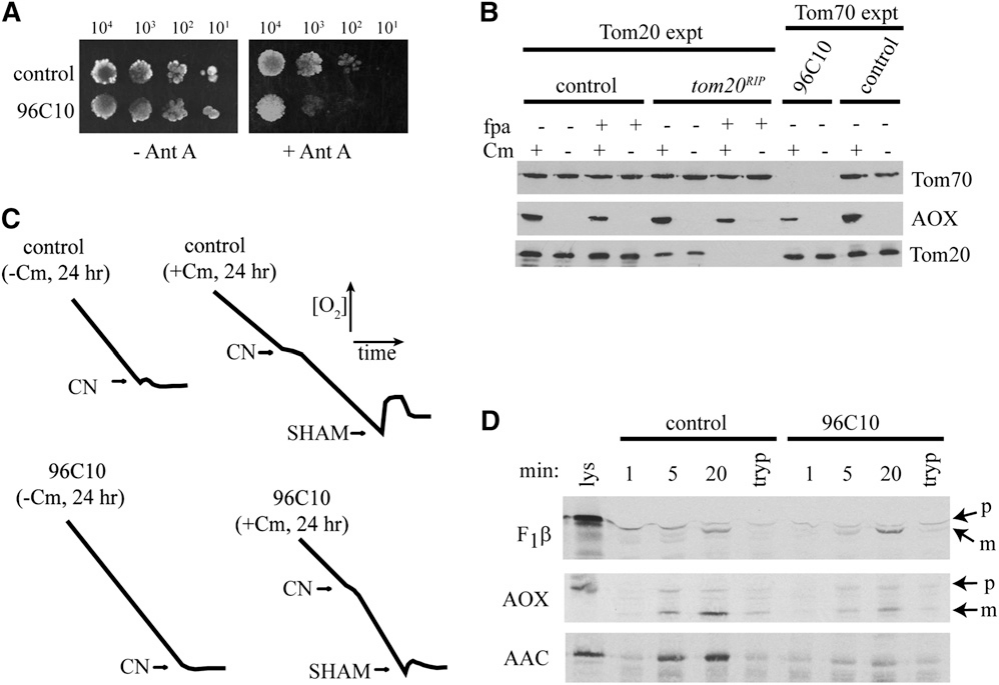
Tom70 is the major receptor for AOX import into mitochondria. (A) The indicated number of conidia from the control (NCN251) and strain 96C10 were spotted onto medium without (−Ant A) or with (+Ant A) antimycin A. Plates were photographed after 2 d (−Ant A) or 4 d (+Ant A) of growth. (B) The indicated strains were grown in liquid medium containing inhibitors as shown. Mitochondria were isolated and subjected to SDS-PAGE followed by Western blot analysis. Antibodies used are indicated on the right. Controls were strain HIV for the Tom20 experiment and NCN251 for the Tom70 experiment. (C) Respiration measurements were taken as in Figure 3B. (D) Mitochondria isolated from the control (NCN251) and 96C10 were incubated with radiolabeled precursor proteins of F_1_β, AOX, and AAC. After the indicated times of incubation, mitochondria were treated with proteinase K to remove unincorporated precursor proteins. Mitochondria were then washed and subjected to SDS-PAGE. The gel was transferred to nitrocellulose and exposed to x-ray film. Lys, 33% of the radiolabeled lysate used in each lane; p, precursor form of the protein; m, mature form of the protein after removal of the targeting signal; tryp, mitochondria were treated with trypsin to remove surface receptors before the addition of labeled precursor to demonstrate that the import observed in other lanes was dependent on mitochondrial surface receptors.

To validate the effects of the *tom70* knockout on AOX, we investigated the phenotype of the strain in more detail. Although we saw no growth of the strain on antimycin A containing medium after 48 hr (Table 1), slow growth was detectable after four d (Figure 4A). We then examined the steady-state levels of AOX protein in mitochondria lacking Tom70 or depleted for Tom20. Because Tom20 is an essential protein (Harkness *et al.* 1994a), we could not study a knockout strain. Therefore, we produced mitochondria with greatly reduced levels of Tom20 from a previously constructed *tom20^RIP^* sheltered heterokaryon (Harkness *et al.* 1994a). Growth of the heterokaryon in the presence of fpa results in numerical superiority of the nucleus that is devoid of a functional *tom20* gene resulting in a reduction of the Tom20 protein in the mitochondria of the culture (See Methods). We isolated mitochondria from cultures of the *tom70* knockout strain (96C10), a Tom20-depleted strain, and appropriate controls. All strains were grown both in the presence and absence of Cm. Mitochondrial proteins were subjected to SDS-PAGE and analyzed on Western blots for the presence of various mitochondrial proteins. Growth of the *tom20^RIP^* sheltered heterokaryon in the presence of fpa resulted in mitochondria virtually devoid of Tom20, while Tom70 levels were unaffected (Figure 4B, Tom20 expt, *tom20^RIP^* lanes). Levels of AOX in these mitochondria after growth in the presence of fpa and Cm were reduced compared with AOX in *tom20^RIP^* cultures grown without fpa. However, the levels of AOX in the *tom20^RIP^* cultures are virtually identical to the control cells grown under the corresponding conditions (Figure 4B, Tom20 expt +fpa +Cm lanes for control and *tom20^RIP^*).

Thus, fpa appears to have an unknown effect in reducing AOX levels, but the presence or absence of Tom20 does not result in changes in AOX levels relative to the control. On the other hand, mitochondria isolated from the *tom70* knockout strain have levels of Tom20 similar to the control strain, but have much reduced AOX levels compared with the relevant control (Figure 4B, Tom70 expt, +Cm lanes), consistent with the results in Figure 2B for 96C10. Respiration measurements on *tom70* knockout cells grown in the presence of Cm showed that the reduced amount of AOX in the cells was still sufficient to allow CN-insensitive respiration to occur (Figure 4C). We assume that the reduced level of AOX in the mitochondria of *tom70* knockout cells grown in Cm is sufficient to allow slow growth in the presence of antimycin A as seen in Figure 4A.

We also directly measured import of radiolabeled precursor proteins into isolated mitochondria with reduced levels of Tom70 (Figure 4D). AOX import was obviously reduced in mitochondria lacking Tom70, as was import of AAC a protein known to depend on Tom70 for its import (Ryan *et al.* 1999; Söllner *et al.* 1990; Young *et al.* 2003b). In contrast, import of the precursor of the β subunit of the F_1_ portion of the mitochondrial ATP synthase (F_1_β), a protein that contains a cleavable N-terminal targeting signal, occurred at a slightly higher level in the mitochondria lacking Tom70. For two quantified experiments, the percentage of import into the mitochondria lacking Tom70 was 100% and 123% of the control for F_1_β; 39% and 47% for AAC; and 35% and 48% for AOX. The simplest interpretation of these data is that Tom70 is the major receptor involved in the import of the AOX precursor. However, we cannot formally eliminate the possibility that another unknown factor that is decreased in mitochondria lacking Tom70 may play a role in AOX import.

## Discussion

This study has identified many genes that are required to achieve full expression of AOX under conditions that induce the enzyme in *N. crassa*. Absence of many of the genes has only minor effects on AOX (class 3), whereas loss of others has a more pronounced effect (classes 1 and 2). Many of the knockout strains identified in our screen exhibit reduced growth rates and/or reduced ability to form conidia. Thus, such genes are not likely to be specific for an AOX regulatory pathway. Loss of these genes may affect several functions that include AOX expression. More information on AOX expression at the transcriptional and posttranscriptional levels, as well as the assembly of the enzyme into the mitochondria inner membrane, will be required to understand the effects in each case.

Although the AOX precursor protein contains a cleavable N-terminal targeting signal, its efficient import appears to be dependent on Tom70. Tom70 was previously shown to enhance the import of a few precursors containing N-terminal cleavable targeting signals (Hachiya *et al.* 1995; Hines *et al.* 1990; Hines and Schatz 1993; Schlossmann *et al.* 1994). A detailed investigation of precursors of this class revealed that their presequences were recognized by Tom20. However, the mature portions of these precursor proteins were prone to aggregation, which could be prevented by association with Tom70 (Yamamoto *et al.* 2009). It was suggested that the requirement for Tom70 is related to its known function as a docking site for cytosolic chaperone proteins (Fan *et al.* 2011; Young *et al.* 2003b), which would likely be associated with aggregation prone precursor proteins. Furthermore, a chaperone activity of Tom70 toward at least some precursors was also identified (Yamamoto *et al.* 2009). Thus, the AOX with its hydrophobic domains that interact with the inner mitochondrial membrane (Andersson and Nordlund 1999; Berthold *et al.* 2000; Berthold and Stenmark 2003) fits nicely into this model of Tom70 function.

Six genes were identified that are predicted to be transcription factors. Two of these, AOD2 and AOD5, were previously characterized as required for AOX production. However, it is now clear that these proteins are involved in the transcription of other genes as well. It has been shown in *P. anserina* that the orthologs of AOD2 and AOD5 (RSE-2 and RSE-3, respectively) are involved in transcription of AOX but also play a role in transcription of genes involved in gluconeogenesis (Sellem *et al.* 2009). Similarly, the *A. nidulans* orthologs (AcuM and AcuK, respectively) are involved in gluconeogenesis and AOX regulation (Hynes *et al.* 2007; Suzuki *et al.* 2012). Interestingly, the *Aspergillus fumigatus* orthologs also are involved in iron uptake (Liu *et al.* 2010). The other transcription factors identified here, may also have multiple roles, including possible contributions to AOX transcription.

We also identified six genes that encode putative kinases or phosphatases. Phosphorylation/dephosphorylation events are well known to be associated with effects on protein function and gene expression. Perhaps the most interesting gene in this category is the Snf1 kinase, which is disrupted in strain 13G11. The *S. cerevisiae* Snf1 kinase and its mammalian ortholog, AMPK, are known to play roles in sensing cellular energy levels (Hardie 2007; Turcotte *et al.* 2010; Young *et al.* 2003a). When activated by conditions resulting in reduced ATP levels, the kinase acts to phosphorylate transcription factors involved in expression of genes for processes such as gluconeogenesis and the glyoxylate cycle. In fact, the Snf1 kinase is involved in phosphorylation of Rds2 (Soontorngun *et al.* 2007), the *Saccharomyces cerevisiae* ortholog of *N. crassa* AOD2 (Chae *et al.* 2007b). Rds2 is involved in gluconeogenesis and increased Snf1-dependent phosphorylation of the protein is observed during growth in ethanol as compared to growth in glucose. Binding of Rds2 at the promoter for the FBP1 gene encoding fructose-1,6-bisphophatase, was dependent on Snf1 activity (Soontorngun *et al.* 2007). Interestingly, the Snf4 gene (30F5) that encodes a homolog of a regulatory factor of the *S. cerevisiae* Snf1 kinase complex also was identified in our screen as a class 3 mutant. Thus, it will be of interest to determine if phosphorylation of AOD2 by *N. crassa* SNF1 plays a role in activation of the protein as a transcription factor of the *aod-1* gene. For *A. nidulans*, it was recently suggested that malate may be a ligand that binds to the PAS domains of either AcuM (AOD2) and/or AcuK (AOD5) to activate the heterodimeric transcription factor (Suzuki *et al.* 2012). Because deletion of the *snf1* gene in *N. crassa* does not completely eliminate AOX expression, it is conceivable that both phosphorylation by SNF1 and binding of malate may be involved in AOD2 activation.

Reactive oxygen species (ROS) are thought to play a role in mitochondrial biogenesis and in eliciting stress responses in many systems (Bae *et al.* 2011; Collins *et al.* 2012; Leister 2012; Rhoads and Vanlerberghe 2004; Yoboue and Devin 2012). Increased levels of ROS are known to be associated with increased levels of AOX in certain fungi such as *C. albicans* (Huh and Kang 2001), *Magnoporthe grisea* (Yukioka *et al.* 1998), and *A. fumigatus* (Magnani *et al.* 2007). On the other hand, this does not appear to be the case for *A. nidulans* (Pusztahelyi *et al.* 2011; Suzuki *et al.* 2012) or *P. anserina* (Borghouts *et al.* 2001). Our screen detected only two genes with an obvious relationship to cellular redox conditions. Superoxide dismutase, which converts superoxide to molecular oxygen and hydrogen peroxide, is inactivated in the class 3 strain 101D3, which has a minor AOX reduction after 48 hr growth in Cm. The specific protein (NCU09560.5) is predicted to encode a version of the enzyme that uses manganese as a cofactor, a hallmark of mitochondrial versions of the enzyme (Holley *et al.* 2010) and the protein was identified as a *Neurospora* mitochondrial protein in a proteomic study (Keeping *et al.* 2011). Because there is only a slight reduction of AOX in the strain after growth in Cm, it would appear that the signaling mechanism for AOX transcription is largely unaffected. Given that AOX is present in this strain during growth in Cm, it is not obvious why it does not grow in the presence of antimycin A (Table 1). One possibility is that the signaling mechanism for AOX production is different for growth in antimycin A *vs.* Cm. Another possibility is that the increased ROS that is likely produced upon antimycin A inhibition proves toxic to cells in the absence of the mitochondrial superoxide dismutase.

The gene removed in the class 2 mutant 85H5, is listed at the genome database as a thioredoxin that also has a glutaredoxin domain (NCU09803.5). BLAST searches against the *S. cerevisae* proteome demonstrated that NCU09803.5 is most highly related to the monothiol glutaredoxins, Grx3 and Grx4 (supporting information, Table S1). These yeast proteins, as well as NCU09803.5, contain a thioredoxin-like domain at their N-terminus, but also the CGFS motif that defines monothiol glutaredoxins (Herrero and De La Torre-Ruiz 2007; Lillig *et al.* 2008; Meyer *et al.* 2009). The latter domain replaces the CXXC motif found in classical thioredoxins and dithiol glutaredoxins.

A search of the *N. crassa* database identified a GRX5 protein that represents a mitochondrial monothiol glutaredoxin and revealed that NCU09803.5 is the only non-mitochondrial form of the protein in the organism (see Table S1). This is similar to *Schizosaccharomyces pombe*, which contains only a single Grx3/Grx4 ortholog, called Grx4 (Chung *et al.* 2005). These proteins are known to play important roles in various aspects of iron metabolism, including iron sensing, influencing the localization and activity of iron responsive transcription factors, and general intracellular iron trafficking (Lill *et al.* 2012; Rouhier *et al.* 2010). With respect to our study, it is of interest to note that depletion of both Grx3 and Grx4 in *S. cerevisiea* has been shown to result in severe reduction in activity of di-iron enzymes in both the cytosol and mitochondria (Mühlenhoff *et al.* 2010). Thus, the defect in expression of AOX, a di-iron carboxylate enzyme, seems likely to be related to iron metabolism in the 85H5 strain. The observation that 85H5 does not grow in the presence of antimycin A even though substantial levels of AOX accumulate after 48 hr growth in Cm may reflect an accumulation of AOX protein without inserted iron.

Three class 2 mutants encode components of the COP9 signalosome (CSN). The CSN is a highly conserved complex in eukaryotes consisting of eight subunits in higher organisms, with some variation in the number of subunits in lower eukaryotes. The CSN is best known for its role in the regulation of protein degradation, but other activities, including possible direct roles in gene expression and regulation of transcription factors, have been associated with the complex or its subunits (reviewed in Chamovitz 2009, Wei and Deng 2003, and Wei *et al.* 2008). In *N. crassa*, study of a *csn-2* disruption strain revealed a role for CSN in the regulation of the circadian clock (He *et al.* 2005). An in depth study of the *N. crassa* CSN complex revealed that it contains seven different subunits: CSN1 through CSN6 plus CSN7a (Wang *et al.* 2010). With the exception of CSN3, cells lacking any of the seven subunits had pleiotropic phenotypes, including defects in proteosome regulation, growth, conidiation, and circadian rhythms. Our screen identified knockouts of the *csn-1*, *csn-2*, and *csn-5* genes as having effects on AOX production (Table 1). At the time we examined the library the *csn-3* and *csn-4* gene knockouts also were present but were not identified in the screen. Interestingly, an *A. nidulans* mutant lacking CSN5 (CsnE) was found to exhibit a complex phenotype that included alterations in redox regulation (Nahlik *et al.* 2010). Furthermore, an interaction between CSN5 (Jab-1) and the antioxidant protein thioredoxin that affects the activity of the AP-1 transcription factor was seen in human cells (Hwang *et al.* 2004). Thus, the effects observed on AOX expression in the *csn-5* knockout in our study may be the result of altered redox regulation. It will be interesting to determine whether the effect of the *csn* gene knockouts on AOX abundance in *N. crassa* is due to an effect on transcription or a posttranscriptional process. There is considerable evidence in other systems suggesting that the CSN2 and CSN5 proteins have roles as individual proteins in gene expression, unrelated to their roles in the complex (Chamovitz 2009; Wei *et al.* 2008).

Our study has revealed several new genes that affect AOX production. It is valid to ask whether we have found any genes that should be named *aod* (AOX deficient). Because our previously named *aod-2* and *aod-5* genes were thought to be AOX-specific transcription factors but are now known to be involved in gluconeogenesis as well (described previously), it may be difficult to assign *aod* to any gene in the absence of further data regarding the exact function of the gene products. The requirement for many different genes to achieve the full level of AOX in mitochondria suggests that complex pathways of expression exist for the enzyme. Closer examination of the mutants identified here will help to elucidate the nature of these mechanisms.

## Supplementary Material

Supporting Information
