# Supplementary material for: Identification of Genes Required for Alternative Oxidase Production in the Neurospora crassa Gene Knockout Library
Source: G3 (Bethesda). 2012 Nov 1;2(11):1345–56. doi: 10.1534/g3.112.004218 (PMC3484665; doi:10.1534/g3.112.004218)
Supplement: Supporting Information [file supp_2_11_1345__index.html]

Supporting Information 

# Identification of Genes Required for Alternative Oxidase Production in the *Neurospora crassa* Gene Knockout Library

## Supporting Information for Nargang *et al.*, 2012

**Files in this Data Supplement:**

- Table S1 - Homologues of NCU09803.5 in the *N. crassa, S. cerevisiae*, and *S. pombe* genomes (PDF, 52 KB)
