# Supplementary material for: Identification of Genes Required for Alternative Oxidase Production in the Neurospora crassa Gene Knockout Library
Source: G3 (Bethesda). 2012 Nov 1;2(11):1345–56. doi: 10.1534/g3.112.004218 (PMC3484665; doi:10.1534/g3.112.004218)
Supplement: Supporting Information [file supp_2.11.1345_TableS1.pdf]

Table S1 Homologues of NCU09803.5 in the *N. crassa*, *S. cerevisiae*, and *S. pombe* genomes

Protein	E value in BLAST to NCU09803
S. cerevisiae GRX3	1.1e-49
S. cerevisiae GRX4	2.0e-48
S. cerevisiae GRX5	1.9e-22
N. crassa NCU04098 (GRX5)	7.3e-21
S. pombe GRX4	2.3e-47
S. pombe GRX5	2.1e-21
